# Diagnostic value of chest ultrasound in children with cystic fibrosis – Pilot study

**DOI:** 10.1371/journal.pone.0215786

**Published:** 2019-07-10

**Authors:** Lidia Strzelczuk–Judka, Irena Wojsyk–Banaszak, Aleksandra Zakrzewska, Katarzyna Jończyk–Potoczna

**Affiliations:** 1 Department of Pediatric Radiology, Chair of General and Invasive Radiology, Poznan University of Medical Sciences, Poznań, Poland; 2 Department of Pulmonology, Pediatric Allergy and Clinical Immunology, Poznan University of Medical Sciences, Poznań, Poland; Cleveland Clinic, UNITED STATES

## Abstract

Cystic fibrosis (CF) is one of the most common genetic disorders among the White population. The disease has a progressive course and leads to a reduction in the quality of life and of life expectancy. Standard diagnostic procedures used in the monitoring of CF patients include methods which expose patients to ionizing radiation. With increasing life expectancy in CF the cumulative dose of ionising radiation increases, prompting clinicians’ search for safer imaging studies. Despite its safety and availability lung ultrasound (LUS) is not routinely used in the diagnostic evaluation of CF patients. The aim of the study was to evaluate the diagnostic value of LUS in children with CF compared to a chest X-ray, and to assess the diagnostic value of the recently developed LUS score—CF-USS (Cystic Fibrosis Ultrasound Score). LUS was performed in 48 CF children and adolescents aged from 5 to 18 years (24 girls and 24 boys). LUS consisted of the assessment of the pleura, lung sliding, A-line and B-line artefacts, "lung rockets", alveolar consolidations, air bronchogram and pleural effusion. Chest radiography was performed in all patients and analyzed according to the modified Chrispin-Norman score. LUS was analyzed according to CF-USS. The correlation between the CF-USS and the modified Chrispin-Norman scores was moderate (R = 0.52, p = 0.0002) and strong in control studies. In 75% of patients undergoing LUS, small areas of subpleural consolidations were observed, which were not visible on x-rays. At the same time, LUS was not sensitive enough to visualize bronchial pathology, which plays an important role in assessing the progression of the disease. Conclusions: LUS constitutes an invaluable tool for the diagnosis of subpleural consolidations. CF-USS results correlate with the conventional x-ray modified Chrispin–Norman score. LUS should be considered a supplementary radiographic examination in the monitoring of CF patients, and CF-USS may provide clinicians with valuable information concerning the progression of the disease.

## Introduction

Cystic fibrosis (CF) is one of the most common autosomal recessive hereditary life-shortening disorders among the White population [1,2]. The disease is caused by the mutation of gene coding CFTR protein (*Cystic Fibrosis Transmembrane Conductance Regulator*), leading to the production of dense mucus in the airways and exocrine glands and the impairment of their functioning. The main affected systems comprise respiratory and digestive systems, and the chronic pulmonary disease remains the main cause of morbidity and one of the most important prognostic factors in CF [1,3,4]. Chronic inflammation due to impaired mucocilliary clearance and mucus impaction in the airways results in bronchiectasis and progressive lung tissue destruction [5].

The lung evaluation in CF patients traditionally involves imaging studies and among these, the most common remains chest x-ray. Early on in the course of disease a radiologic picture might reveal no abnormalities. Along with the progression of the disease lung hyperinflation and increased bronchial markings appear, followed by chest infiltrates, atelectasis and bronchiectasis [1,4].

The need for objective tools in the evaluation of patients has prompted the development of x-ray scoring systems, including the Brasfield score [6] the Northern score [7] the Chrispin-Norman score [8] and its modified version [9]. These scoring systems are used for the monitoring of disease progression, evaluation of different therapies as well comparison of patients’ outcomes between the treatment centres [4,7,8–14].

The most accurate radiographic diagnostic modality in CF, the so-called”golden standard” that allows for qualitative and quantitative evaluation of lung involvement, even very early on in the course of the disease remains computed tomography (CT) [4]. CT, due to its high resolution allows the visualisation of the details that are not visible in a plain chest x-ray [10]. In CF patients, CT enables the visualisation of the bronchial wall and of peribronchial thickening, intralobular nodules, bronchiolitis, the so-called”tree in bud” sign, air trapping, bronchiectasis, mucus impaction, microabscesses, infiltrates, atelectasis, enlarged lymph nodes and the widening of pulmonary artery, along with narrowing of peripheral vessels [5,15,16]. The role of CT in CF patients was confirmed in studies reporting the correlation of the CT scans with patients outcomes [17]. For a quantitative, objective evaluation of CT results in CF patients scoring systems were also developed with the most popular Bhalla score [18].

The disadvantage of CT scanning is a the relatively high dose of ionising radiation. The risks of cancer related to the total amount of radiation that the patient was exposed to during his/her lifetime made clinicians look for imaging modalities with the lowest or ideally no radiation [19]. Ultrasound (US) is currently one of the most important and most frequently used imaging techniques [20]. With this in consideration, lung ultrasound (LUS) as a safe, non-invasive, widely available and cheap technique might constitute an important tool in the diagnostic protocols of children with CF [21]. Despite this fact, there are few existing reports on the application of LUS in CF patients. There are only two reports published as abstracts by Ciuca et al on LUS in CF as compared to CT scans [22,23].

A LUS examination comprises the evaluation of the pleural line and lung sliding [24–28], an analysis of the artefacts that are present in a normal lung, such as “the bat sign” [24,25,29,30] and the A-line artefacts [25,28,31,32] as well as those in pathological conditions (the B-line, Z-line and I-line artefacts) and the evaluation of thoracic wall structures. The B-lines are vertical, well defined hyperechogenic lines, arising from the pleural line, spreading out without fading to the edge of the screen, similar to a laser beam or a”comet tail” artefact [25,33,34]. Multiple B-lines are typical for interstitial lung disease [35–37]. Seen together they are described as the”lung rockets” artefact [28,32,37]. Multiple coalescent B-lines in the absence of A-lines with visible lung sliding constitute the so-called”white lung” image [37–39]. Alveolar consolidation can be diagnosed with LUS only if these consolidations are localised in the peripheral part of the lungs and according to the literature reports this is the case in up to 98.5% of all cases [24,29,32]. The aim of our report was to evaluate the diagnostic value of the chest ultrasound in children with CF as compared to the plain x-ray, as well as to assess the diagnostic value of the recently developed LUS score—CF-USS (Cystic Fibrosis Ultrasound Score).

## Material

We enrolled 48 patients of European descent (24 males) aged 5 to 18 years diagnosed with CF who were admitted to the Pulmonology Department for a scheduled annual diagnostic check-up. The patients underwent a chest ultrasound and plain x-ray, and the time interval between the studies was not longer than 72 hours.

In all the children and adolescents studied CF was confirmed by two positive sweat test results and genetic studies (two pathogenic mutations). All the patients and their parents gave informed consent for the study. Parents or guardians and patients 16 years old and older gave written informed consent, and younger children verbal informed consent.

Exclusion criteria comprised severe immunosuppression, a lack of consent and a time interval longer than 72 hours between the studies. The study design was accepted by the Bioethical Committee of Poznań University of Medical Sciences. In Table 1 we presented the characteristic of the patients studied.

**Table 1:** Patients characteristic.

Number of patients	48	
Males/females	24 / 24	
	Mean ± SD	Median (minimum-maximum)
Age (years)	11.9 ± 3.9	12 (5–18)
BMI	16.7 ± 2.9	15.7 (10.2–24.2)
FEV_1_ (L)	1.9 ± 0.8	1.7 (0.4–4.3)
BMI <3c N(%)	7 (14.6)	
Pancreatic sufficient N (%)	9 (18.8)	
F508del homozygous N(%)	21 (43.8)	
F508del heterozygous N(%)	18 (37.5)	
Chronically infected with *Pseudomonas aeruginosa*	21 (43.8)	
FEV_1_ ≥ 80% normal value	32 (66.7)	
FEV_1_ ≤ 40% normal value	1 (2.1)	

BMI–Body mass index; FEV_1_ –Forced expiratory volume in the first second; N- number; SD–standard deviation

## Methods

### Radiographic imaging

X-rays were performed with an analogue apparatus Axiom Iconos R 100 (Siemens Healthcare), in posteroanterior projection during suspended inspiration. The technical parameters of the images (including the use of the grid, the source image receptor distance, the dose of radiation) were individually adjusted for every patient studied in concordance with ALARA (As Low As Reasonably Achievable) principle, in order to achieve the best possible images using the lowest radiation dose. No lateral x-rays were performed. X-rays were independently evaluated by two board—certified paediatric radiologists with experience in CF. Chest x-rays were evaluated using the modified Chrispin-Norman score [9].

### Chest ultrasound (LUS)

A chest ultrasound was performed with iU22 apparatus (Philips, Biothel United States) using a linear probe of 5–12 mHz (L12-5) frequency and, depending on the patients’ age, with either a convex probe of 1–5 mHz (C5-1) frequency, a convex probe of 4–9 mHz (C9-4) frequency or a microconvex probe of 5–8 mHz (C8-5) frequency through longitudinal and transverse sections of the anterior, lateral and posterior walls of the chest. The preliminary preset was soft tissue, excluding artefact reduction options (SonoCT, XRes). Doppler imaging was used for the evaluation of vascularisation of the inflammatory changes.

The patients were examined in the sitting position. A chest ultrasound was performed by applying the probe to the anterior, lateral and posterior surfaces of the chest. Transverse sections of the chest wall were obtained by the transverse application of the probe and scanning the whole available area in the cranio-caudal direction. Longitudinal sections were obtained by applying the probe along the parasternal line, the midclavicular line, the anterior axillary line, the midaxillary line, the posterior axillary line, the scapular line and the paravertebral line moving the probe along the intercostal spaces. The studies were performed by two board certified paediatric radiologists with experience in LUS and CF.

In every patient we evaluated respectively: the quality (free flowing or organised, localization) and quantity (fluid layer in millimetres) of any fluid present in the pleural space, the shape and thickness of the pleural line, the lung sliding sign, A-lines and B-lines artefacts (their number, localisation and morphology, including single ones as well as”lung rockets” complexes and “white lung” images) and alveolar consolidations (their number, dimensions, localisation, morphology, presence of bronchogram and its characteristic (air or fluid) and vascularisation).

LUS results were classified according to the scoring system developed by the authors: CF-USS *(Cystic Fibrosis Ultrasound Score)* devised on the basis of the modified Chrispin-Norman score and the bronchiolitis score reported by Caiulo and collaborators [39,40]. Scores are calculated separately for the anterior (between the sternum and the midaxillary line) and the posterior (between the midaxillary line and the spine), the surface of the right and left half of the thorax moving the probe in the cranio–caudal direction and along the intercostal spaces, obtaining longitudinal and transverse sections. Each part can be scored from 0 to 2 points for irregularities of the pleural line, single and complex B-line artefacts, alveolar consolidations and the presence of fluid in the pleural space, with a maximum score of ten for each part, and 40 in total. The higher the score, the more advanced the disease process is (Table 2).

**Table 2:** Cystic fibrosis ultrasound score.

Characteristic	Intensity		
**Pleural irregularities**	**Absent**	**Present**	**Present+ pleural thickening**
Right lung: anterior surface	0	1	2
Right lung: posterior surface	0	1	2
Left lung: anterior surface	0	1	2
Left lung: posterior surface	0	1	2
**Focal B-line artefacts**	**Absent /few (≤6)**	**Some (7–14)**	**Many (≥15)**
Right lung: anterior surface	0	1	2
Right lung: posterior surface	0	1	2
Left lung: anterior surface	0	1	2
Left lung: posterior surface	0	1	2
**Coalescent B-line artefacts**	**Absent**	**Fused**	**„Lung rockets”**
Right lung: anterior surface	0	1	2
Right lung: posterior surface	0	1	2
Left lung: anterior surface	0	1	2
Left lung: posterior surface	0	1	2
**Subpleural consolidations**	**Absent (≤6)**	**Some (7–14)**	**Multiple or extensive (≥15)**
Right lung: anterior surface	0	1	2
Right lung: posterior surface	0	1	2
Left lung: anterior surface	0	1	2
Left lung: posterior surface	0	1	2
**Pleural fluid**	**Absent**	**Obliterating phreno–costal angle**	**In phreno–costal angle and along the chest wall**
Right lung: anterior surface	0	1	2
Right lung: posterior surface	0	1	2
Left lung: anterior surface	0	1	2
Left lung: posterior surface	0	1	2

### Statistical analysis

Statistical analysis was performed with Statistica software (version 12; StatSoft). Data distribution was evaluated with the Shapiro-Wilk test. For data with normal distribution we used t Student test for paired and independent variables. For data that did not meet the normal distribution assumptions, Spearman’s rank correlation coefficient was calculated. P value of <0.05 was considered statistically significant.

## Results

### Comparison of LUS and chest x-ray images

**Fig 1:**
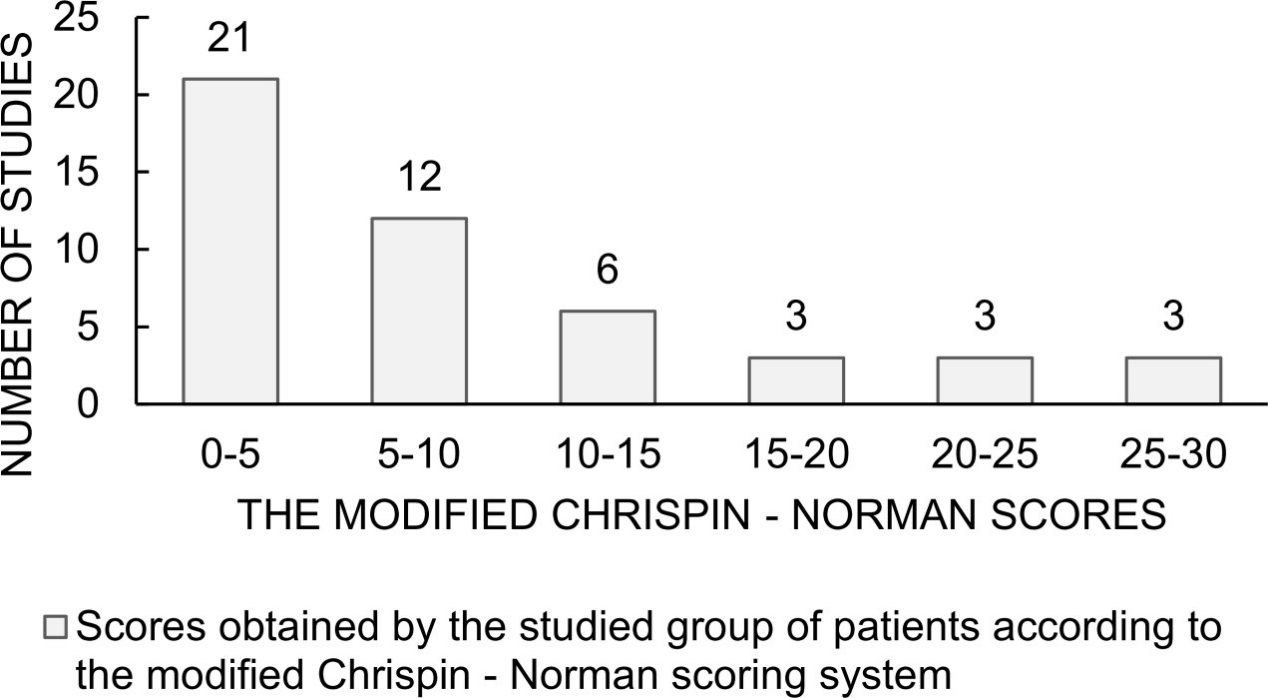
The modified Chrispin-Norman scores. Scores obtained by the patients studied in the modified Chrispin–Norman scoring system.

**Fig 2:**
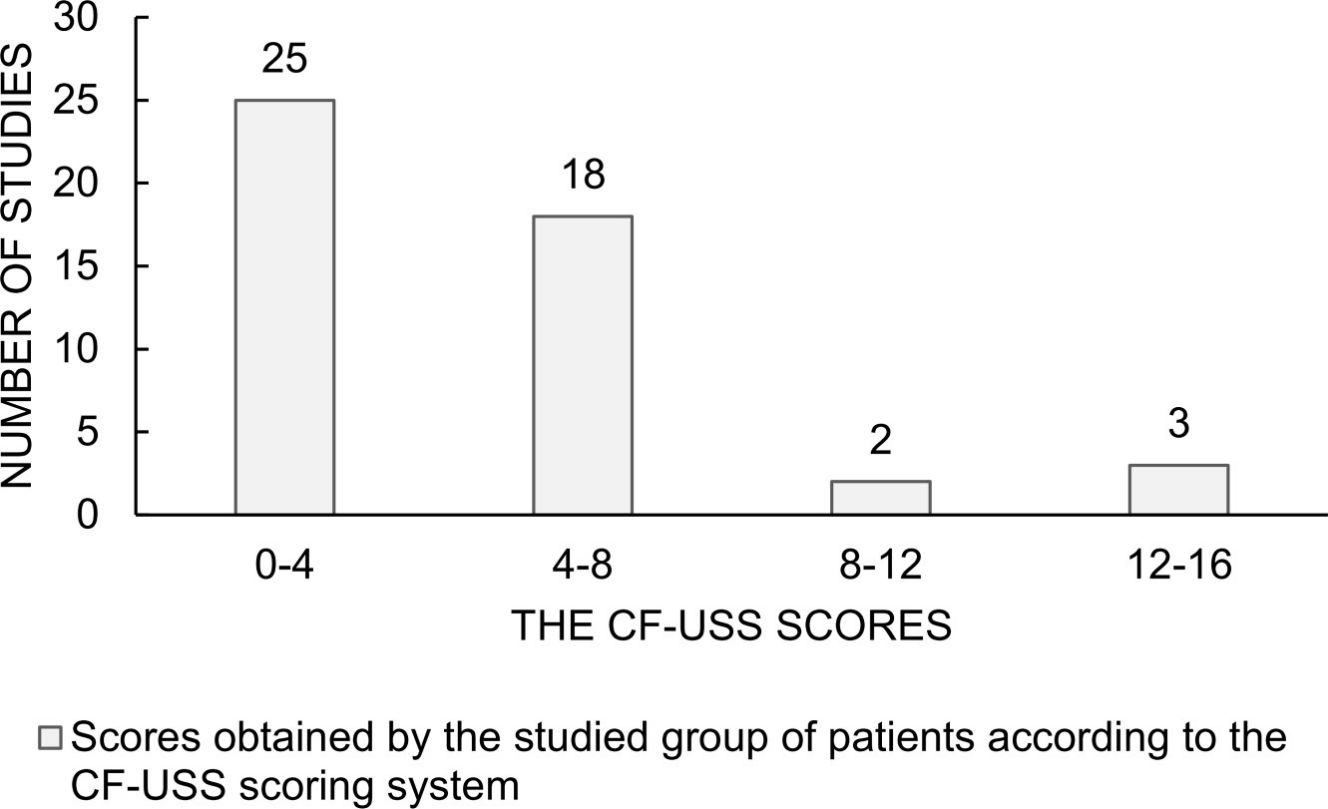
The CF-USS scores. Scores obtained by the patients studied in the CF-USS scoring system.

**Fig 3:**
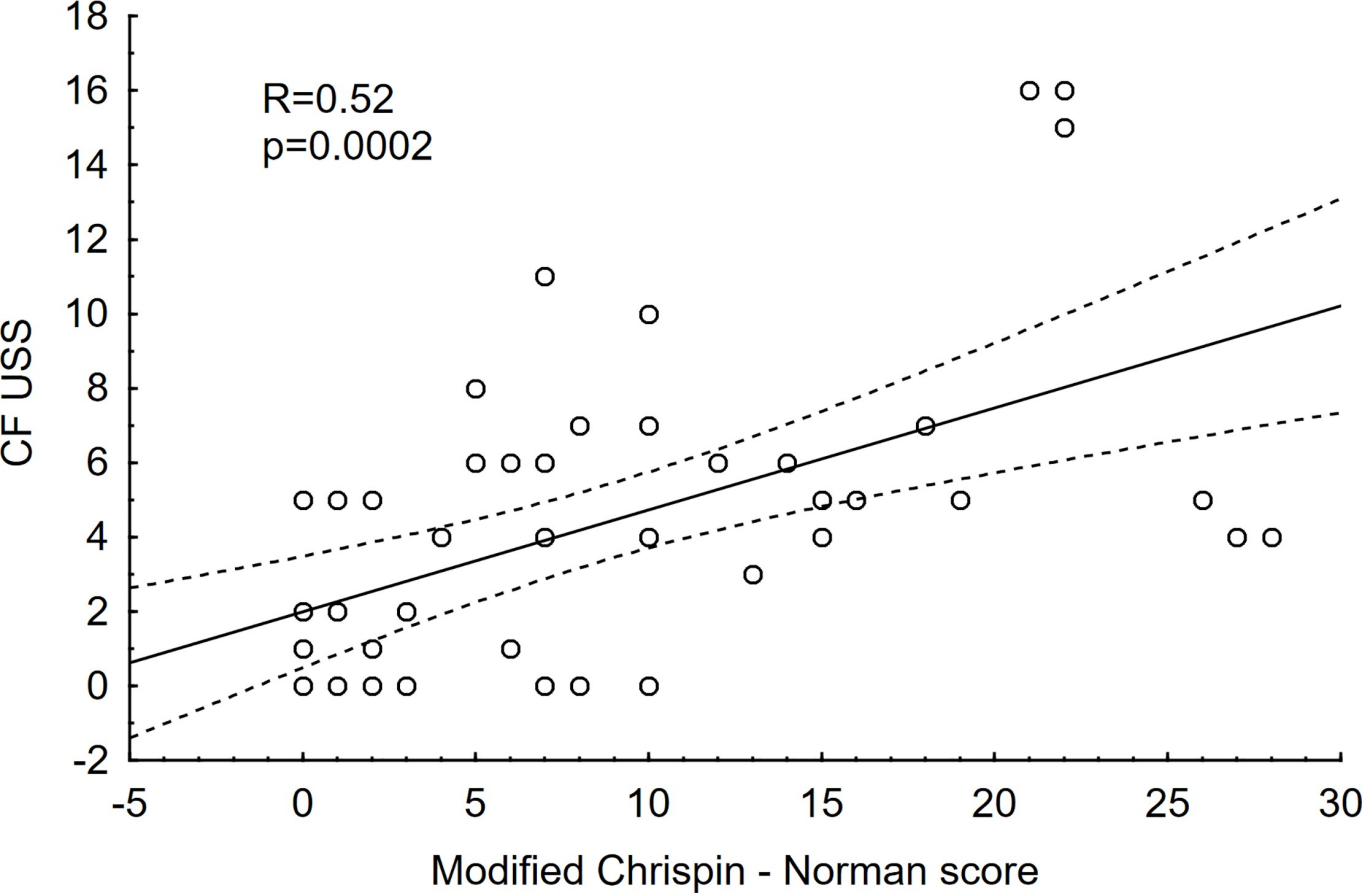
Correlation between CF-USS and the modified Chrispin-Norman scores. R- Spearman’s rank correlation coefficient; p–p value; CF-USS—scores obtained by the patients studied in the CF-USS scoring system; modified Chrispin–Norman score—scores obtained by the patients studied in the modified Chrispin–Norman scoring system.

Pulmonary disease was evaluated radiologically with the modified Chrispin–Norman scoring system for the x-rays and the CF-USS score. The patients’ results are shown in Table 3 and Figs 1 and 2. The statistical analysis has shown positive correlation between the two scoring systems (R Spearman = 0.52, p = 0.0002) (Fig 3).

**Table 3:** Comparison of the two scoring systems.

Parameter	Modified Chrispin–Norman score	CF-USS
Number of patients	48	48
Mean ± SD	8.7 ± 8.0	4.4 ± 4.1
Median (minimum-maximum)	7 (0–28)	4 (0–16)

CF-USS–Cystic fibrosis ultrasound score; SD–standard deviation

Fine subpleural consolidation were seen in LUS in 36 patients (75%). Abnormalities seen in LUS in the patients studied and classified according to CF-USS are presented in Table 4.

**Table 4:** Number of patients in the group studied with abnormalities according to the CF-USS score.

Characteristic	Number of patients	Percentage (%)
**Pleural irregularities**	1	2
**Focal B-line artefacts–**some	33	69
**Focal B-line artefacts—**many	4	8
**Fused B-line artefacts–**coalescent	19	40
**Fused B-line artefacts -** „Lung rockets”	1	2
**Subpleural consolidations–**few	28	58
**Subpleural consolidations—**multiple or extensive	8	17
**Pleural fluid—**in costo–phrenical angle	9	19
**Pleural fluid—**and along the chest wall	2	4

**Fig 4:**
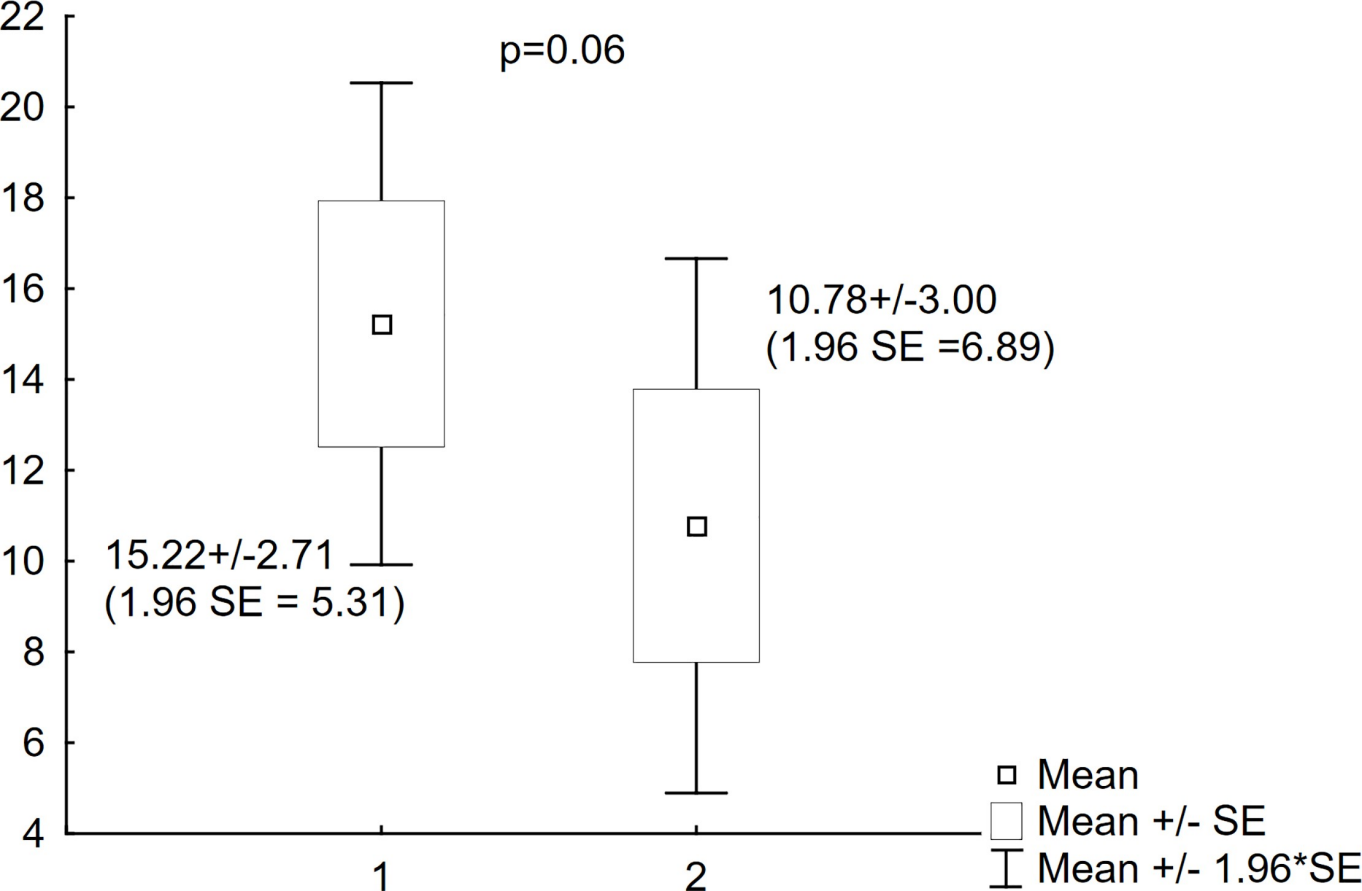
Modified Chrispin–Norman scores for the repeated studies. Comparison of scores obtained in the modified Chrispin–Norman scoring system on two different occasions. 1. Modified Chrispin–Norman scores of the first study; 2. Modified Chrispin–Norman scores of the repeated study.

**Fig 5:**
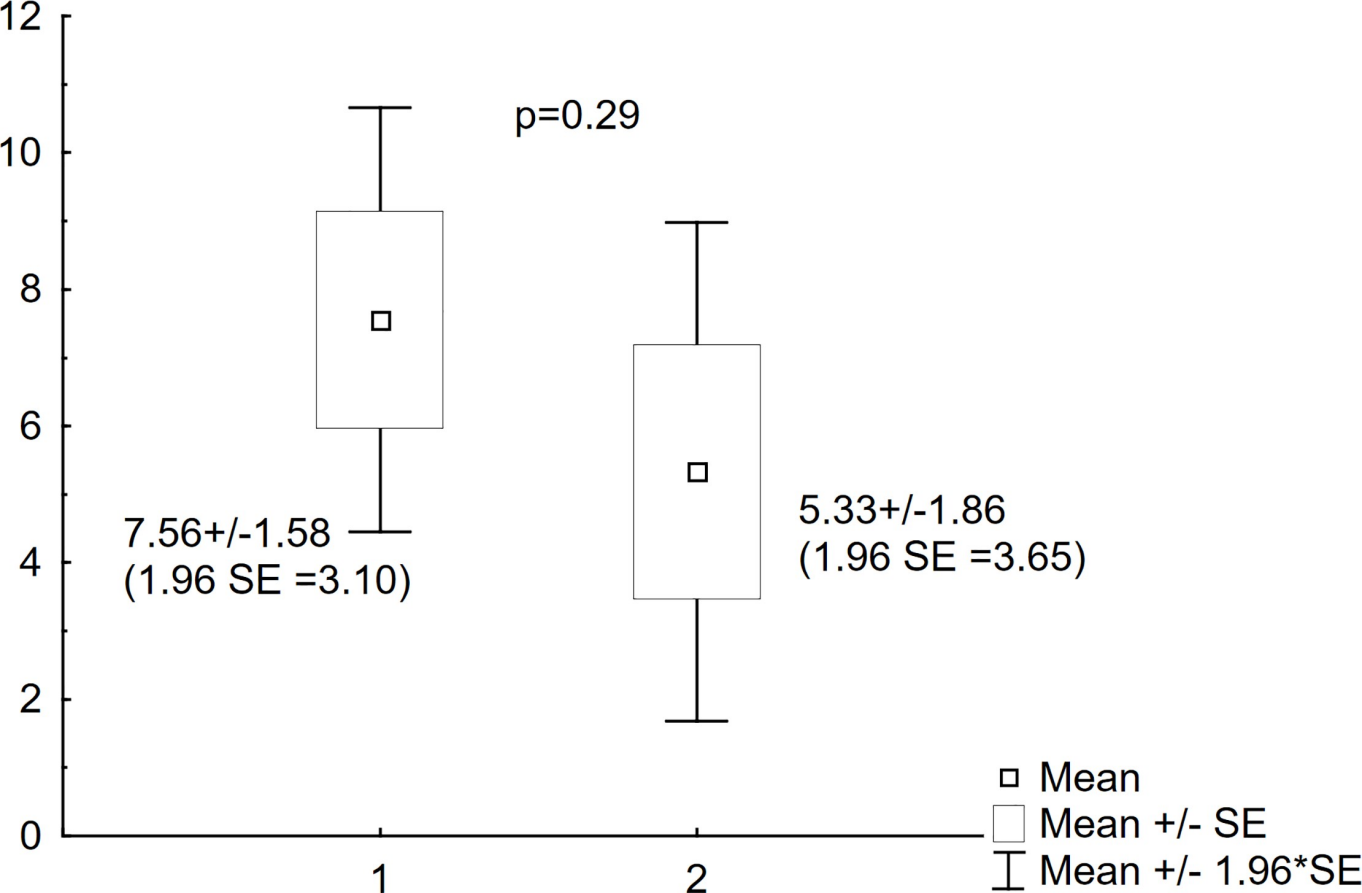
CF-USS scores for the repeated studies. Comparison of scores obtained in the CF-USS scoring system on two different occasions. 1. CF-USS scores of the first study; 2. CF-USS scores of the repeated study.

**Fig 6:**
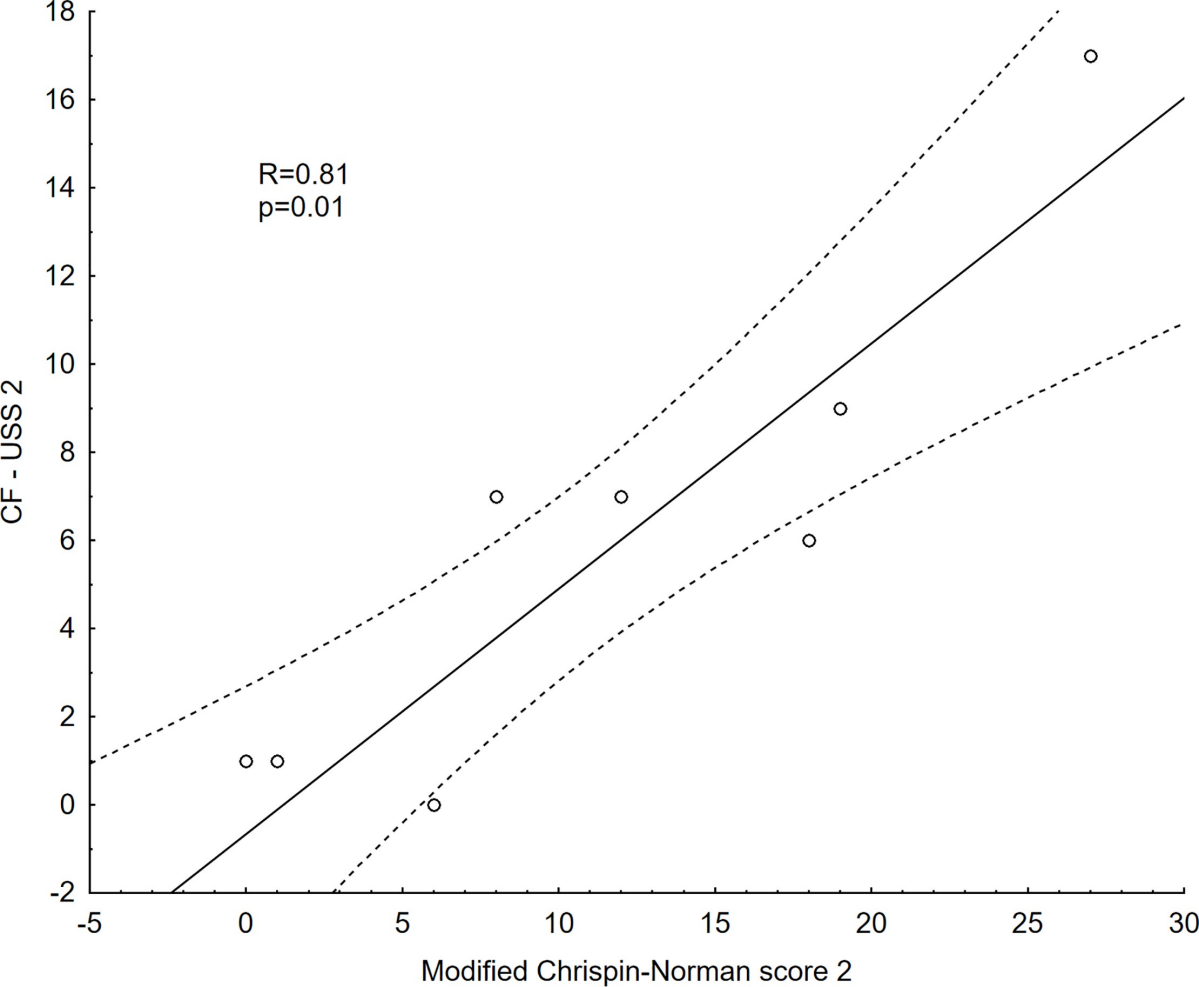
Correlation between the CF-USS score and the modified Chrispin-Norman score in the repeated studies. R- Spearman’s rank correlation coefficient; p–p value; CF-USS—scores obtained by the patients studied in the CF-USS scoring system in the repeated studies; modified Chrispin–Norman score—scores obtained by the patients studied in the modified Chrispin–Norman scoring system in the repeated studies.

In nine patients LUS and x-ray were performed twice on two different occasions. There were no statistically significant differences between the x-rays in the modified Chrispin–Norman score (15.22±2.71 vs. 10.78±3.00; p = 0.06) and LUS in CF-USS score (7.56±1.58 vs. 5.33±1.86; p = 0.29) (Figs 4 and 5). Statistical analysis for the repeated LUS and x-ray examinations showed a positive correlation for the two studies (R Spearman = 0.81, p = 0.01) (Fig 6).

**Fig 7:**
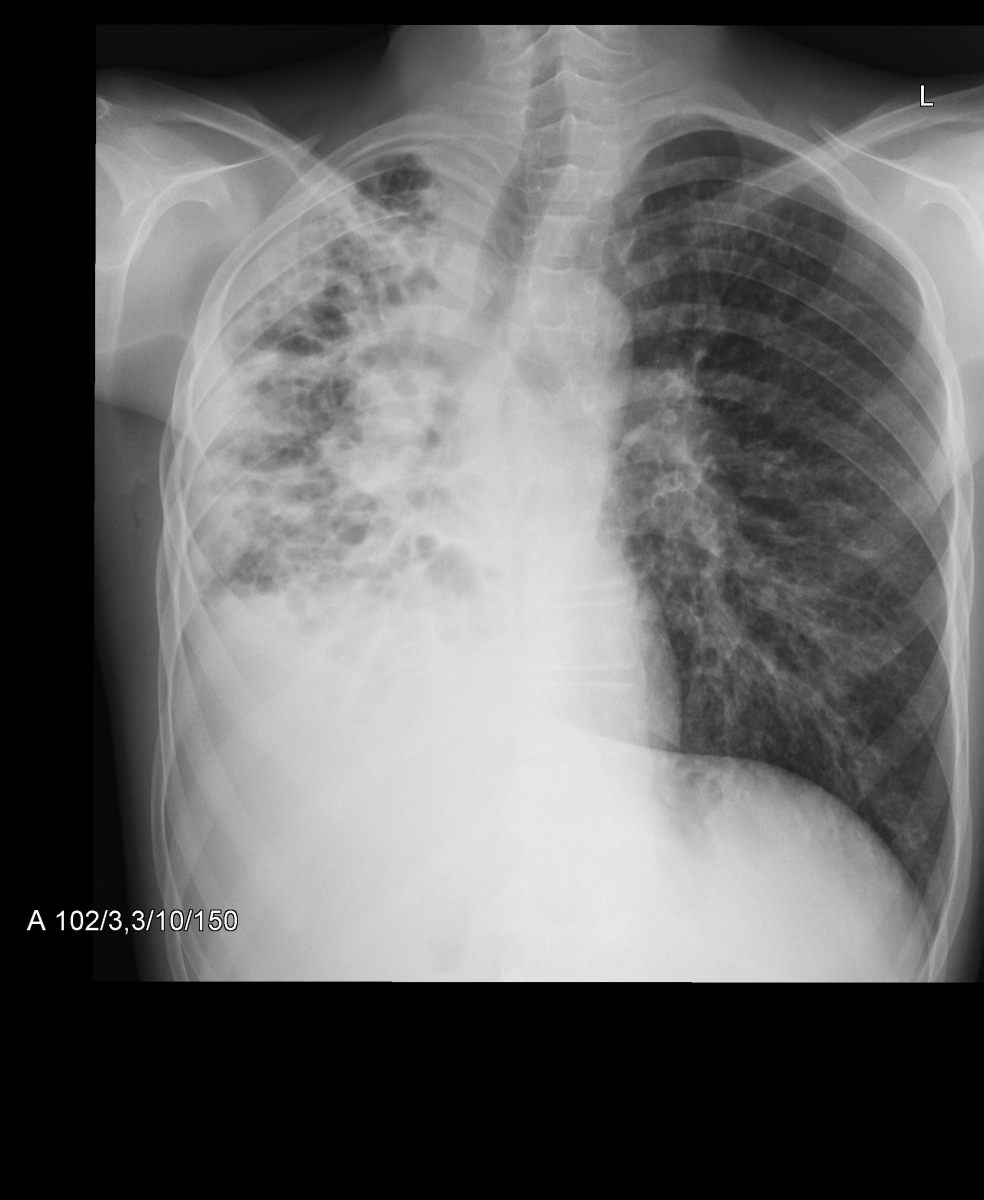
Mediastinal shift, atelectasis and pleural effusion on the right, linear and cystic opacities, bronchiectasis, consolidations. Hyperinflation of the left lung, with nodular and linear interstitial opacities. 27 points in the modified Chrispin-Norman score.

**Fig 8:**
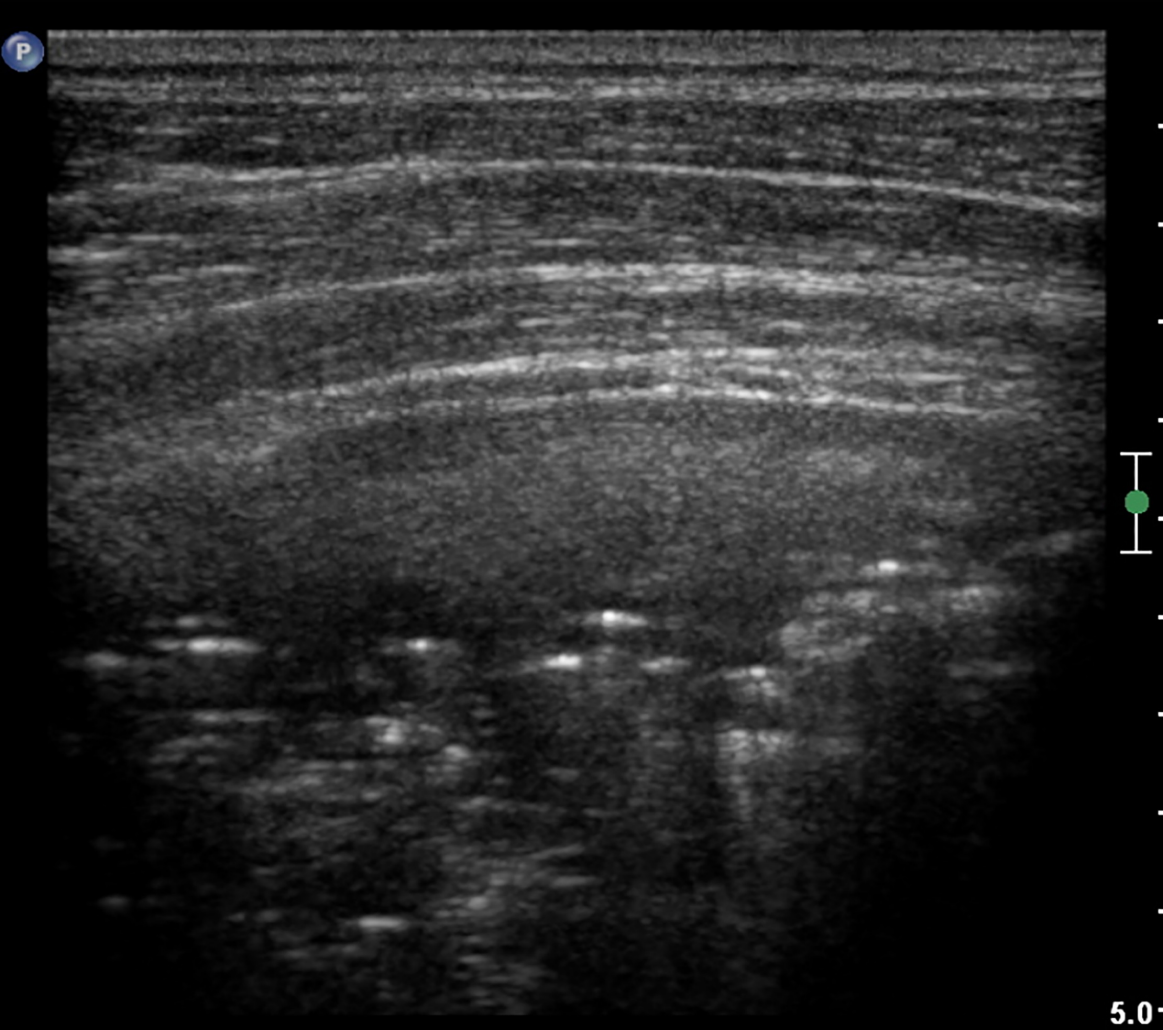
LUS image of the patient from Fig 7, linear probe. Regions of consolidation and atelectasis. 17 points in the CF-USS.

**Fig 9:**
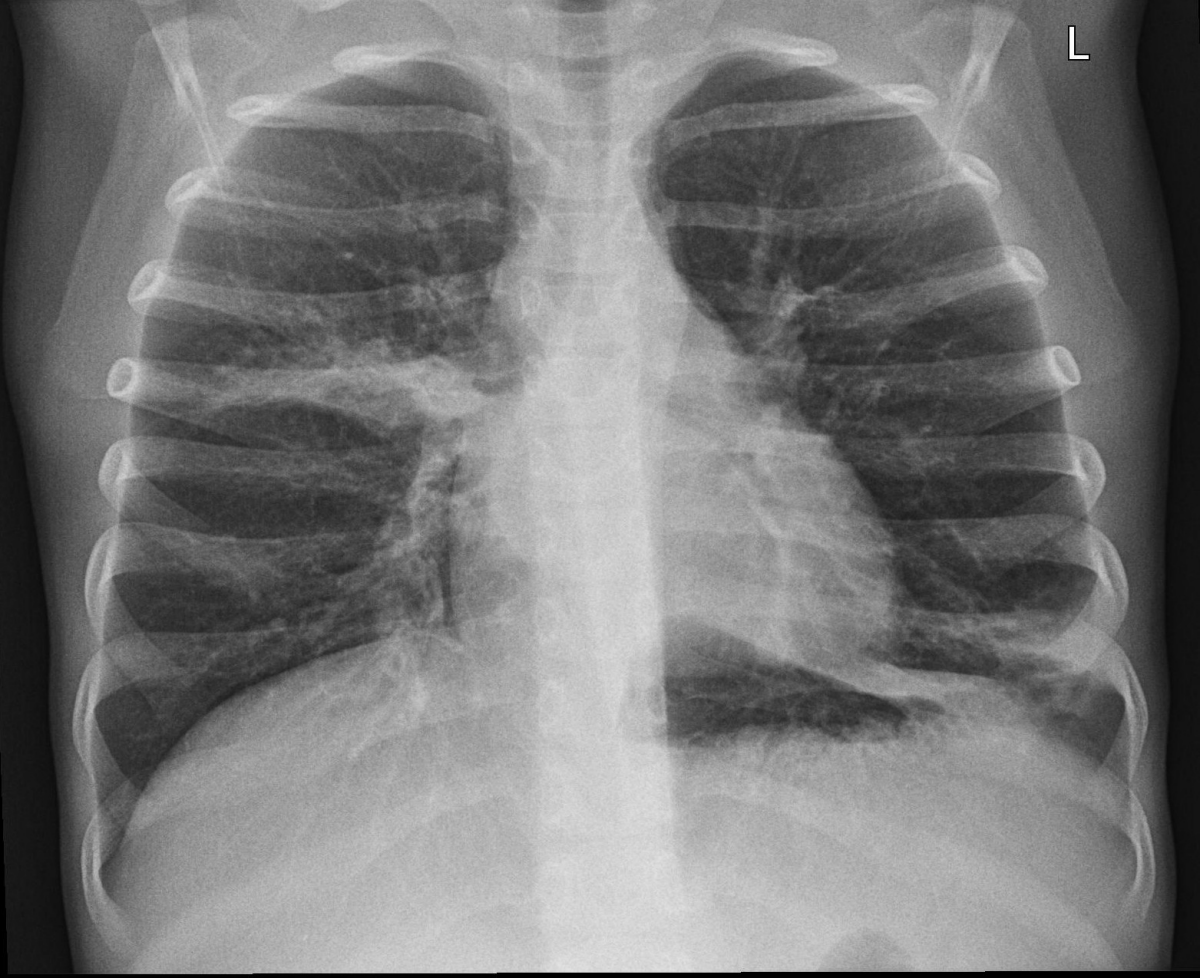
Linear opacities and regions of consolidations in the middle right and lower left field. Fine peribronchial infiltrates in the lower right and middle left field. Hyperinflation of both lungs. 15 points in the modified Chrispin-Norman score.

**Fig 10:**
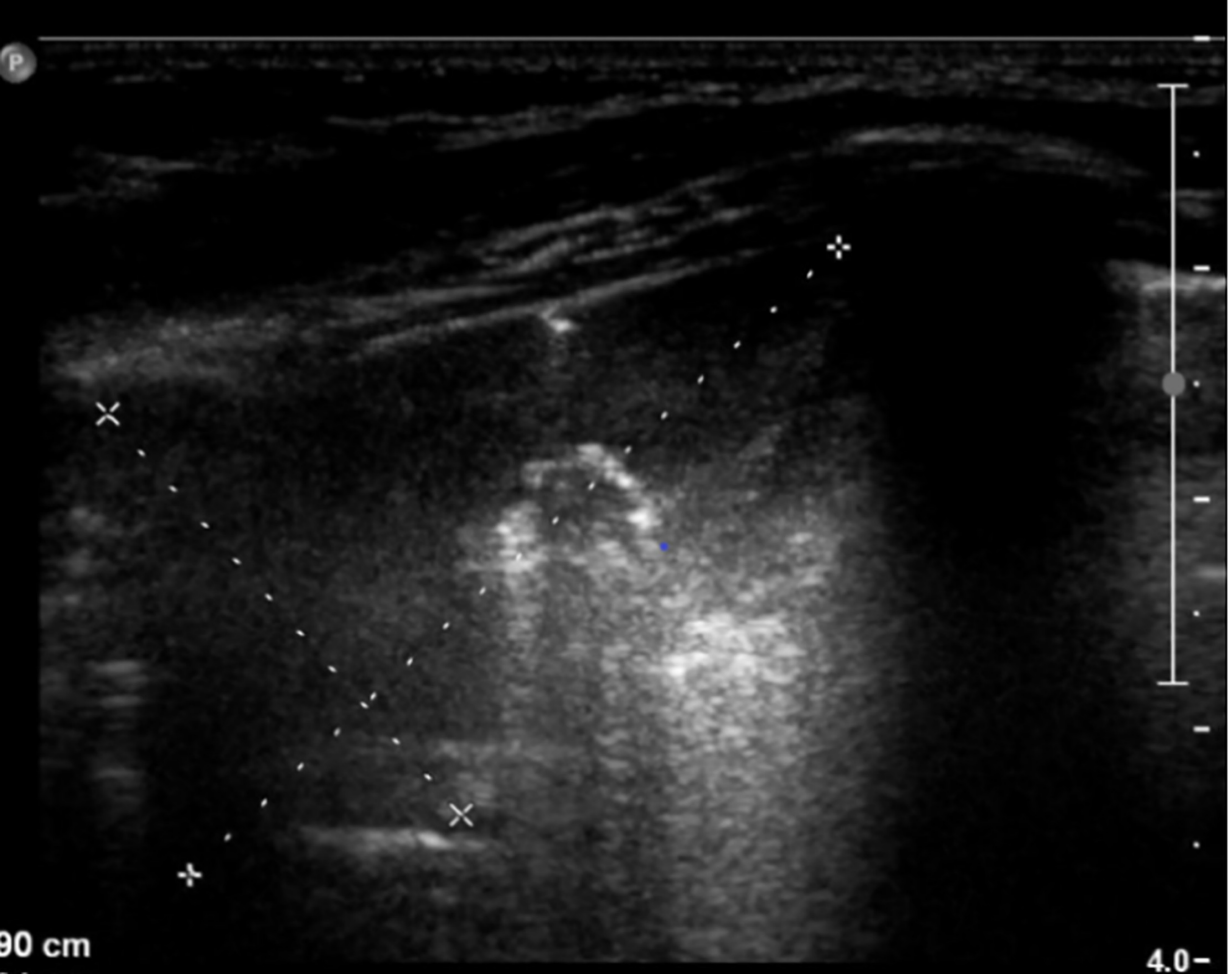
LUS image of the patient from Fig 7, linear probe. Large area of consolidation in the right upper lobe. 5 points in the CF-USS.

**Fig 11:**
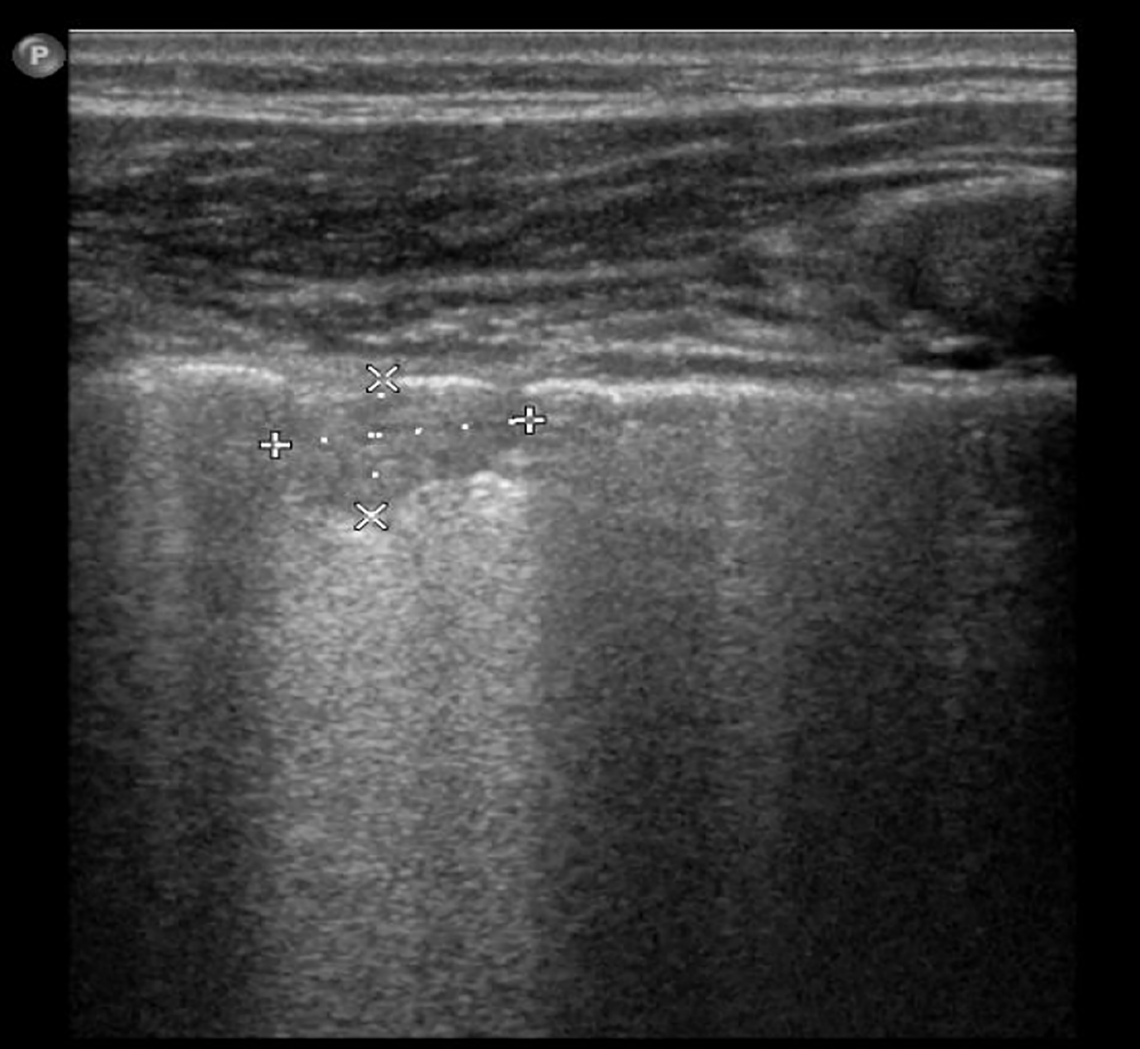
LUS image of the patient from Fig 7, linear probe. Fine subpleural consolidation.

In the Figs 7–11 chest x-ray and LUS images of the studied patients are presented.

## Discussion

Cystic fibrosis is a life-shortening genetic disorder, involving the respiratory system and requiring chronic therapy. During the course of the disease patients suffer recurrent exacerbations, that affect patients quality of life and survival. Radiology plays a significant role in the patients’ follow-up, enabling monitoring of the disease, response to treatment as well as the diagnosis of exacerbations [41]. Unfortunately conventional x-rays, especially when they are numerous and repeated over the course of disease, add up to a cumulative ionising radiation dose. Diminishing the radiation exposure, by looking for alternative diagnostic modalities, should be considered as one of the goals of contemporary medicine.

A chest ultrasound has not been routinely used in the monitoring of lung disease in paediatric patients with CF, despite its lack of radiation, availability and safety. Considering this we wanted to compare the diagnostic value of LUS with conventional chest x-rays assessed according to the modified Chrispin–Norman score. There are no other studies comparing the modified Chrispin–Norman score with a chest ultrasound in CF paediatric patients. Furthermore, we developed our own ultrasound scoring system (CF-USS) to make the comparison more feasible.

For the evaluation of the chest x-ray we chose the modified Chrispin–Norman score as it uses only antero–posterior projections for the evaluation of the hyperinflation of the chest based on the shape of the thorax, diaphragm location and lung hyperlucency resulting from air-trapping [9]. This remains in alignment with the data from Benden et al. and allows for a diminished radiation dose while avoiding the lateral projection [9]. Terheggen-Lagro and colleagues, in their study, compared six different clinical and radiological scoring systems (the Schwachman–Kulczycki score, the Chrispin-Norman score, the modified Chrispin-Norman score, the Brasfield score, the Wisconsin score and the Northern score) and demonstrated their clinical utility in different clinical settings [14]. The authors proved, that radiographic scoring systems in the CF patients, especially the modified Chrispin-Norman score are characterized by low interobserver variability and correlate with pulmonary function tests results as well as clinical features.

The aim of this study was to compare the results of the x-ray scoring system with the chest ultrasound scoring system. Furthermore we constructed a novel chest ultrasound score for the evaluation of CF paediatric patients (CF-USS). The score has been developed based on the experience of Caiulo and colleagues, who used LUS in patients with bronchiolitis, which pathology, among others, is also present in CF patients [5,15,40,42].

LUS was performed at the same time as the chest x-ray. In nine patients LUS was performed twice on two different occasions. The most commonly seen pathological features were B-line artefacts of different numbers and intensities. B-line artefacts might be seen in a normal lung and are not considered pathological as long as their number does not exceed 2 in a single transverse scan with a convex probe and 6 in a single longitudinal scan with a high resolution linear probe [32].

The clinical relevance of the B-line artefacts is quite wide and has recently been covered in an excellent review by Dietrich and colleagues [43]. The authors believe, that B-line artefacts can be caused by multiple factors, and can be present in lung oedema, heart failure, lung interstitial diseases, infections, acute respiratory distress syndrome (ARDS) or lung injury. B-line artefacts are the sign of an increased lung density, due to the loss of the lung tissue aeration. Chiesa and colleagues found B-line artefacts in 37% of elderly studied as compared to 10% of healthy young adults [44]. A correct B-line artefacts interpretation should account for the evaluation of other LUS signs and clinical data. In pathological conditions B-line artefacts may be useful for the monitoring of treatment. The influence of technical factors on the appearance of B-line artefacts still remains to be elucidated [43].

In our LUS scoring system (CF-USS) the B-line artefacts are divided into focal (few, some and many) and coalescent (absent, fused and “lung rockets”). The scoring system reflects the intensity and the variability of the lung pathology, known as B-line artefacts. Despite a statistically significant correlation between the two scoring systems studied in our material, we believe that true clinical significance of B-line artefacts, in a single LUS examination without clinical data, has important limitations. In the children with CF, B-line artefacts should be evaluated in the context of the progression of the disease, documenting their numbers and localisation [45].

Another pathology seen in LUS are subpleural consolidations. Very fine 3 to 4 mm in diameter subpleural consolidations may be present in healthy children in the first few years of life [20,46]. In children with CF, however, they have important clinical implications. Dense mucus blocks the airways, including the bronchiole, leading to focal atelectasis and hyperinflation, and resulting in recurrent infections. Peripheral mucus plugs cause small foci of inflammation, that might progress into disease exacerbations [10]. There are several studies illustrating the fact that structural changes seen in radiologic examinations may be seen ahead of pulmonary function deterioration [47]. In CF-USS, subpleural consolidations were classified as: absent, few and multiple or extensive. In 75% of the patients studied subpleural consolidations were seen in LUS and in 17% the changes were multiple or wider than 10 mm. None of the changes smaller than 10 millimetres were seen in conventional radiograms. In our opinion, subpleural consolidations, similarly to B-line artefacts, cannot be evaluated without clinical data. Brody and colleagues reported that in stable CF patients subpleural consolidations should be monitored, as they may lead to the clinical deterioration and decline in pulmonary function test results [47]. Subpleural consolidations may increase in dimension and cause patients’ deterioration. Therefore monitoring patients frequently with LUS enables a close follow-up in those with fine subpleural changes, allowing for an earlier diagnosis of exacerbations. The number of LUS examinations is limited only by the number of follow-up visits so the patients are monitored more closely and, at the same time, with greater safety limiting the number of x-rays performed and in consequence radiation exposure.

CF-USS also comprised the evaluation of pleural fluid which seems reasonable to perform in patients suspected of pleural complications regardless of conventional x-ray results. In the group studied in 23% of patients we have documented the presence of fluid in the pleural space, the majority of them having just a small amount in the costo–phrenical angle. Pleural irregularities were seen in only one of the patients studied. Caiulo and colleagues reported finding, in their bronchiolitis study, pleural irregularities in 25% of the patients that disappeared in the course of follow-up [40]. The reason for the disparity of our results might be the fact, that bronchiolitis is not present in all patients with cystic fibrosis.

In 9 patients we repeated LUS and conventional x-rays in a stable condition in the course of a period of 2 years’ follow-up. There were no statistically significant differences between either the LUS or the x-rays. Spearman rank correlation coefficient between x-ray and LUS was higher in the second series of studies.

We believe that LUS is an important diagnostic tool, supplementary to conventional x-rays enabling the monitoring of the process of the disease. We do acknowledge, however, the limitations of the CF-USS scoring system. Our material is small and the study was conducted in a single CF centre–we do hope, however, that the study will be continued in the future. The most important limitation remains the inability of visualisation of consolidations separated from pleura as well as of the airway damage (bronchiectasis, mucus plugs) that constitute the mainstay of lung pathology in CF pulmonary disease. Nevertheless, due to its safety, non-invasiveness and availability we trust that CF-USS will find its place in the long-term monitoring of the disease, the response to treatment and for identifying patients at risk of exacerbations.

## Conclusions

1. LUS should be supplementary radiographic examination in scheduled follow-up visits in cystic fibrosis paediatric patients, and the CF-USS scoring system may provide clinicians with valuable information concerning progression of the disease.
2. The CF-USS results correlate with the conventional x-ray modified Chrispin–Norman score.
3. LUS constitute an invaluable tool for the diagnosis of subpleural consolidations
4. LUS limitations include the inability to visualise consolidations separated from the pleura and larger airways. The numerous clinical conditions in which B-line artefacts can be present additionally makes it difficult to recommend LUS as the sole diagnostic modality in cystic fibrosis patients.
